# Random domain name and address mutation (RDAM) for thwarting reconnaissance attacks

**DOI:** 10.1371/journal.pone.0177111

**Published:** 2017-05-10

**Authors:** Kai Wang, Xi Chen, Yuefei Zhu

**Affiliations:** Department of Network Engineering, Zhengzhou Information Science and Technology Institute, Zhengzhou, Henan, China; Tongji University, CHINA

## Abstract

Network address shuffling is a novel moving target defense (MTD) that invalidates the address information collected by the attacker by dynamically changing or remapping the host’s network addresses. However, most network address shuffling methods are limited by the limited address space and rely on the host’s static domain name to map to its dynamic address; therefore these methods cannot effectively defend against random scanning attacks, and cannot defend against an attacker who knows the target’s domain name. In this paper, we propose a network defense method based on random domain name and address mutation (RDAM), which increases the scanning space of the attacker through a dynamic domain name method and reduces the probability that a host will be hit by an attacker scanning IP addresses using the domain name system (DNS) query list and the time window methods. Theoretical analysis and experimental results show that RDAM can defend against scanning attacks and worm propagation more effectively than general network address shuffling methods, while introducing an acceptable operational overhead.

## Introduction

One important phase in most cyber attacks is network reconnaissance, where the attacker attempts to gather information about the potential target, including its IP address, operating system, and network services, searching for vulnerabilities to exploit and attack. It has been reported that attackers can spend an average of 45 percent of their time performing reconnaissance [1]. Therefore, mitigating the network reconnaissance phase is an effective network defense strategy.

In conventional networks, the host’s network address and service port remain unchanged throughout the service lifetime, and this static service characteristic gives the attacker sufficient time to scan the target for detailed information and deploy a subsequent attack. However, traditional security systems such as firewalls, intrusion detection systems (IDS), and antivirus software are passive and incomplete in nature, and can detect only known attacks.

Moving target defense (MTD) [2] is a diversity defense that increases the attacker’s costs and risk of detection by constantly changing the attack surface of the target, rendering the information collected by the attacker ineffective. Network address and service port shuffling methods [3–19] are the main MTD approaches to impeding network reconnaissance, by dynamically changing the IP addresses and ports to show the attacker a dynamic network, which can be used in conjunction with other security methods, such as active recovery of network components [20][21][22] and dynamic platform [23][24], to enhance network security and resilience.

Some network address and service port shuffling methods [4–9] need to modify the host’s system or software, which can effectively distinguish between attackers and legitimate users, but these methods also increase the cost of deployment. In this paper, we concentrate our study on network-based dynamic methods that modify network facilities without modifying hosts to achieve a dynamic network. For network-based dynamic methods, limited by the limited address space, network address shuffling cannot effectively defend against random scanning attacks [25]. Although the addition of port shuffling can increase the attacker’s scanning space, if the port shuffling is not transparent to the client host, then the client host’s system and software must be modified to obtain dynamic ports [6]; if the port shuffling is transparent to the client host by mapping outside the client host, then the port shuffling is also transparent to the attacker, who is connected with the client host in the same physical network [7]. Therefore, network address and service port shuffling methods offer limited defense performance. In addition, most network address shuffling methods rely upon the server’s static domain name to establish communication after the network address mutation, where the client host acquires the server’s dynamic IP address by sending a DNS request with the server’s domain name. Therefore, these methods provide no defense against an attacker who knows the target’s domain name.

In this paper, we propose a network defense method called Random Domain name and Address Mutation (RDAM) to increase the defense performance against internal and external reconnaissance and scanning. In RDAM, both the domain name and network address are changed dynamically, and the legitimate client must send a DNS request with the server’s dynamic domain name to acquire the server’s IP address. Therefore, the attacker cannot build a domain-based hit-list, and the attacker has to expend more resources when scanning the domain space because the domain space is much larger than the address space. We evaluate the effectiveness and overhead of RDAM through theoretical analysis and experiment, and the results show that RDAM can effectively defend against scanning attack and worm propagation, while introducing an acceptable operation overhead.

The rest of this paper is organized as follows. Section 2 discusses related works. Section 3 describes the basic principles of RDAM. In Section 4, we describe the basic architecture and communication protocols of RDAM. Section 5 provides the evaluation results regarding the proposed method’s effectiveness and overhead. Section 6 concludes this paper.

## Related works

Network address shuffling techniques aim to achieve proactive network defense by dynamically changing the IP address (and service port) of the target. Currently, researchers have proposed a variety of network address shuffling techniques, in which two patterns emerge: hopping and mutation [3].

In the hopping pattern, both communication sides need to change their addresses synchronously by shared *a priori* knowledge and synchronization scheme; thus, their software or systems need to be modified, which increases the deployment cost. MT6D (Moving Target IPv6 Defense) [4] is a hopping technique in IPv6 proposed by Dunlop et al.; this technique aims to maintain user privacy and protect against targeted network attacks by repeatedly rotating the addresses and ports of both communicating hosts. The hopping in MT6D is transparent to the user and does not interrupt existing connections [5]. MT6D can effectively increase the difficulty of executing a scanning attack because of the large IPv6 space; however, MT6D devices must communicate through a shared symmetric key and a synchronized timestamp, and maintain multiple IPv6 address for each node at any given time. RPAH (Random Port and Address Hopping) [6] is a port and address hopping technique proposed by Dunlop et al.; this method builds port and address hopping tunnels via the address hopping gateway (AHG), port hopping engine (PHG), and port and address hopping gateway (PAHG). This method requires adding several hardware devices in the network and deployment software in server hosts. In addition, in RPAH, the destination port for the client host’s connection to the service remains untouched, and therefore, the port hopping does not increase the scanning space. These researchers also proposed TPAH (TAP-based Port and Address Hopping) [7], in which both communication sides generate port and address pair bases on a shared secret key and time-synchronization information. DYNAT (Dynamic Network Address Translation) [1] and APOD (Applications That Participate in Their Own Defense) [8] use address and port hopping approaches to disguise the host’s identity; NAH (Network Address Hopping) [9] enhances the security of data by transferring data across multiple data connections named channels. These three works aim to resist sniffer attacks, but they cannot defend against scan-based reconnaissance attacks.

In the mutation pattern, address shuffling does not need to be synchronized, and the client host usually connects to the server host by a DNS request/respond or other third-party mechanisms. NASR (Network Address Space Randomization) [10] provides a LAN-level network address randomization scheme against hit-list worms; this method uses dynamic host configuration protocol (DHCP) to reassign addresses, and relies upon DNS to reestablish connection after the DHCP update by querying the updated address with the host’s hostname. This method provides limited unpredictability and mutation speed because the mutation is limited within the network address space and DHCP update reconfiguration. OF-RHM (OpenFlow Random Host Mutation) [11], RHM (Random Host Mutation) [12][13][14], and spatio-temporal address mutation [15] are proposed by Jafarian et al.; OF-RHM provides an IP mutation technique for a software defined network (SDN). In OF-RHM, the SDN controller dynamically assigns each host a random virtual IP which is translated to/from the real IP of the host, and hosts reach each other via the virtual IP acquired from the DNS request. OF-RHM uses the SDN controller to mutate the above virtual addresses and maintain connections by maintaining flow table entries. RHM implements a similar IP mutation technique in traditional networks. In spatio-temporal address mutation, the ephemeral IP address of the host is spatially changed based on the requestor identity and temporally changed based on the time. The above techniques can resist scanning attacks and worm propagation, but they have limited effects on scanning attacks and cannot prevent worms from spreading because of the limited IP address space. In addition, because the above techniques rely on the host’s static domain name for the DNS request/response to establish the connection, they cannot defend against an attacker who already knows the target’s domain name. The same limitations apply to the SDN shuffle approach [16], in which the SDN controller orders the server to install network address translation (NAT) rules to translate the dynamic IP address. The SDN shuffle approach modifies all server hosts and the DNS server to cooperate with the SDN controller. Other network address shuffling techniques that employ the mutation pattern include dynamic honeypot networks [17][18] and MOTAG (MOving Target defense mechanism AGainst Internet denial-of-service attacks) [19], which is proposed against denial-of-service (DDoS) attacks, while our work aims at resisting reconnaissance attacks, increasing the cost to attackers through the dynamic mutation of network addresses and domain names.

Regarding the theoretical analysis of the effectiveness of network address shuffling, Carroll et al. [25] presented probabilistic models for non-repeat scanning in static address and perfect shuffling networks, where perfect shuffling is characterized by uniform shuffling where the change rate is greater than scanning rate. Crouse et al. [26] presented probability models for honeynet based on the above models. Their analysis shows that network address shuffling has a limited defense effect against scanning attacks and that perfect shuffling is beneficial only if the network serves a relatively small number of vulnerable computers. Al-Shaer et al.[12][13] theoretically analyze the probability of non-repeat scanning in the uniform mutation network, considering the ratio of the attacker scanning rate to the defender mutation rate. Likewise, this analysis is also limited to non-repeat scanning. Several other theoretical researches related to network address shuffling include the general theory of MTD [27][28], qualitative evaluations of network-based MTD methods [29], and comparisons of different MTD techniques [30].

## Basic principles of RDAM

In a statically configured network, hosts communicate through static IP addresses, and therefore the attacker can scan the IP address space, identify a target hit-list after receiving responses, and execute persistent attacks using the hit-list. In a network address shuffling network based on domain name mapping, the IP address of the host constantly changes, and therefore the attacker cannot maintain a target hit-list of effective IP addresses. However, the domain name of the host remains static, and therefore the attacker can maintain a domain-based hit-list to attack. In addition, limited by the limited IP address space, network address shuffling cannot effectively defend against random scanning attacks.

RDAM dynamically changes the host’s IP address and domain name based on the SDN architecture. A legitimate client host must first make a DNS request before connecting to the service, and the client can connect to the service only if the correct domain name is requested. The client can retrieve the correct domain names through a security authentication server. Therefore in RDAM, the attacker cannot establish a target list of domain names or addresses, and it is difficult for the attacker to directly access a host in the network through the IP address. While the domain name space is much larger than the address space, RDAM greatly increases the difficulty to an attacker trying to scan the network based on domain names.

## Basic architecture of RDAM

**Fig 1:**
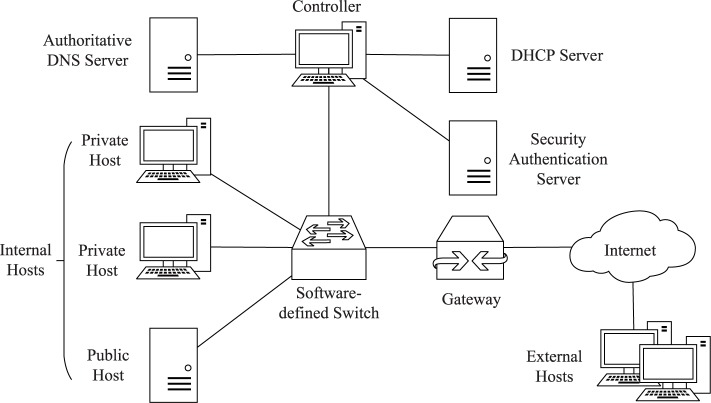
The architecture of RDAM.

Fig 1 shows the basic architecture of RDAM. RDAM uses the SDN architecture, in which all hosts are connected to each other through software-defined switches. The software-defined switches are configured to modify and forward packets quickly; and the controller acts as the central manager of all software-defined switches, managing their flow tables and cooperating with the authoritative DNS server, the DHCP server, and the security authentication server. In RDAM, the authoritative DNS server is authorized for the internal hosts’ domain names, and the security authentication server, which applies existing proof-of-work (PoW) schemes [31][32] to protect the client authentication channel, presents different dynamic domain name lists to authorized users according to their authority.

The internal hosts in RDAM include public hosts that provide services to the external network (Internet) and private hosts that cannot be accessed by the external network. Internal hosts communicate with each other through internal addresses, while public hosts communicate with the external network through external addresses. If private hosts need to access the external network, the gateway must deploy the NAT functionality to realize the communication with the external network.

### 4.1 Address and domain name mutation

In RDAM, the mutation space to be considered includes the internal address space, the external address space, and the domain name space.

Internal addresses and external addresses should be reserved addresses and public addresses in the IPv4 address space, respectively. Denote *S*^*R*^ as all reserved addresses and *S*^*P*^ as all public addresses; then the internal address space is *S*^*I*^ ⊂ *S*^*R*^ with a size *m*^*I*^ = |*S*^*I*^| ≤ 2^24^ + 2^20^ + 2^16^; the external address space is *S*^*E*^ ⊂ *S*^*P*^ with a size (the number of available public addresses) *m*^*E*^ = |*S*^*E*^| that is very limited because of the IPv4 address space allocation strategy.

We use third-level domain (3LD) names for the hosts’ domain names, where the 3LD is a random string of length *l* > 1. Because domain names consist of letters (case-insensitive), numbers, and connectors (not at the beginning or end), then *S*^*D*^ is the domain name space with a size of *m*^*D*^ = |*S*^*D*^| = 36^2^ × 37^*l*−2^. By setting the appropriate *l*, *m*^*D*^ can be much larger than *m*^*E*^ (*m*^*D*^ ≫ *m*^*E*^). In particular, when *l* is 6, the size of the domain name space can be the same order of magnitude as the entire IPv4 address space size.

Suppose there are *n*^*I*^ internal hosts in the RDAM network $Hosts=\left\{h_{1},h_{2},\ldots ,h_{n^{I}}\right\}$, of which there are *n*^*E*^ ≤ *n*^*I*^ public hosts $Servers=\left\{s_{1},s_{2},\ldots ,s_{n^{E}}\right\}$. Denote *mac*_*i*_ as the MAC address of host *h*_*i*_, and *if*_*i*_ as the interface of the software-defined switch to which *h*_*i*_ is connected. In particular, denote *if*^*E*^ as the interface of the software-defined switch to which the gateway is connected, and *GMAC* as the MAC address of the gateway’s interface connecting to the internal network. Then the address and domain name mutation method can be executed as follows.

#### 4.1.1 Internal address mutation

In RDAM, the internal address *iIP*_*i*_ ∈ *S*^*I*^ of host *h*_*i*_ is the host’s real address. In order to protect communications within a physical subnet (a group of hosts physically connected through common switches), the internal addresses of all the hosts in RDAM occupy different subnets of the internal address space so that the internal hosts cannot communicate directly through common switches. Denote *L* as the mask length of all subnets; then the subnet of *iIP*_*i*_ is $S_{i}^{I}=\text{subnet}\;iIP_{i}/L$, and $\forall i\neq j,S_{i}^{I}\cap S_{j}^{I}=\phi$. Therefore, the number of internal hosts supported by RDAM satisfies *n*^*I*^ ≤ *m*^*I*^/2^32−*L*^.

In order to ensure normal communication and confuse the attacker in RDAM, each subnet $S_{i}^{I}$ has a virtual gateway, the IP address of which is $gIP_{i}\in S_{i}^{I}$ and *gIP*_*i*_ ≠ *iIP*_*i*_, and the MAC address *gMAC*_*i*_ is generated randomly. The virtual gateway can respond to some basic protocols such as address resolution protocol (ARP) and Internet control message protocols (ICMP). After the connection to the network has been established, each host will obtain the MAC address of its virtual gateway through ARP protocol for subsequent communications.

The controller can directly obtain DNS requests from internal hosts; therefore in RDAM, for each host *h*_*i*_ we record its internal DNS query list $Q_{i}^{I}\subset S^{I}$, which is the address list of the internal hosts that have queried the given host’s domain name (the address list will be cleared after the domain name changed). Only the connection requests of the hosts in this list are allowed to forward to *h*_*i*_. Therefore, if the attacker does not know the internal host's domain name, then even if the attacker knows the host’s internal address, the attacker still cannot access the host, so the internal addresses can mutate over a long period or remain unchanged.

In addition, the internal host can obtain the internal address through DHCP, and can also use a statically configured internal address; however, the subnet of the configured address cannot be repeated by other internal hosts. If hosts in the same physical subnet are configured with the same subnet, then they can directly access each other without protection. Therefore, in RDAM, all the hosts should be configured in separate subnets.

#### 4.1.2 External address mutation

Because of the recursive query, the controller may not obtain the IP address of the external host in the DNS request from the external network; therefore, it is not possible to identify the external host issuing the DNS query. To address this issue, RDAM uses the time window method to reduce the probability that the attacker can access the public host through the IP address. For each public host *s*_*i*_, we record its external DNS query time $t_{i}^{Q}$, which is the time of the latest DNS query for its external address *eIP*_*i*_ ∈ *S*^*E*^ from the external network. Only within the following *win*_*i*_ time range can external hosts access *s*_*i*_ through *eIP*_*i*_.

In order to calculate the time window of each public host, RDAM counts the number of times each public host is DNS-queried by an external host through its domain name to obtain the average speed *λ*_*i*_(*t*) of the DNS query for *s*_*i*_ at time *t*. It can be assumed that the time interval of external hosts issuing the DNS query for *s*_*i*_ follows an exponential distribution with parameter *λ*_*i*_(*t*). Then the probability that external hosts can access *s*_*i*_ through *eIP*_*i*_ is $p_{i}=1-e^{-\lambda_{i}\left(t\right)win_{i}}$. In RDAM, we set the above probability of each public host as *p*. Then the time window of *s*_*i*_ is $win_{i}=-\frac{1}{\lambda_{i}\left(t\right)}\ln\left(1-p\right)$; that is, the more a public host is DNS-queried by external hosts per unit time, the smaller its time window will be.

In the time window, the attacker could still access the public host through the external address, and therefore the external address of the public host still needs to be dynamically mutated at a high frequency. Denote $R_{i}^{E}$ as the external address mutation rate of public host *s*_*i*_; then the next external address mutation time $t_{i}^{E}$ is the current mutation time plus $1/R_{i}^{E}$. $R_{i}^{E}$ can be pre-configured, and the more critical a public host is, the higher its mutation rate should be.

#### 4.1.3 Domain name mutation

Each host *h*_*i*_ has a dynamic domain name *d*_*i*_ ∈ *S*^*D*^. For the convenience of users, the domain names of all hosts are periodically changed at fixed times. Denote *T*_0_ as the first domain name mutation time, and *T*^*D*^ as the time interval of domain name mutation; then the next domain name mutation time after time *t* is $next\left(t\right)=T_{0}+\left(\left\lfloor \frac{t-T_{0}}{T^{D}}\right\rfloor +1\right)T^{D}$.

Because the domain name space has sufficient size, it can guarantee that in a sufficient number of domain name mutation cycles, a domain name will not be repeated. Assume the non-repeat cycle number of the domain name mutation is *x*; then RDAM records the domain names *D*^*x*^ ⊂ *S*^*D*^ that are assigned in the last *x* cycles. Assume *φ* denotes the maximum ratio of the number of allocated domain names |*D*^*x*^| = *xn*^*I*^ to the domain name space size; then the non-repeat cycle number of domain names supported by RDAM satisfies *x* ≤ min(*m*^*D*^/*n*^*I*^−1,*φm*^*D*^/*n*^*I*^).

### 4.2 Controller algorithm

#### 4.2.1 Packet processing algorithm

In RDAM, software-defined switches modify and forward transmission control protocol (TCP) and user datagram protocol (UDP) packets according to flow tables; packets that have no matching flows in flow tables and ARP, ICMP, DNS, and DHCP packets are forwarded to the controller. The controller processes the above packets and installs the flows necessary for new connections in the software-defined switches in the path. Each connection must be associated with some fixed flows, thus ensuring end-to-end connectivity continuity.

**Fig 2:**
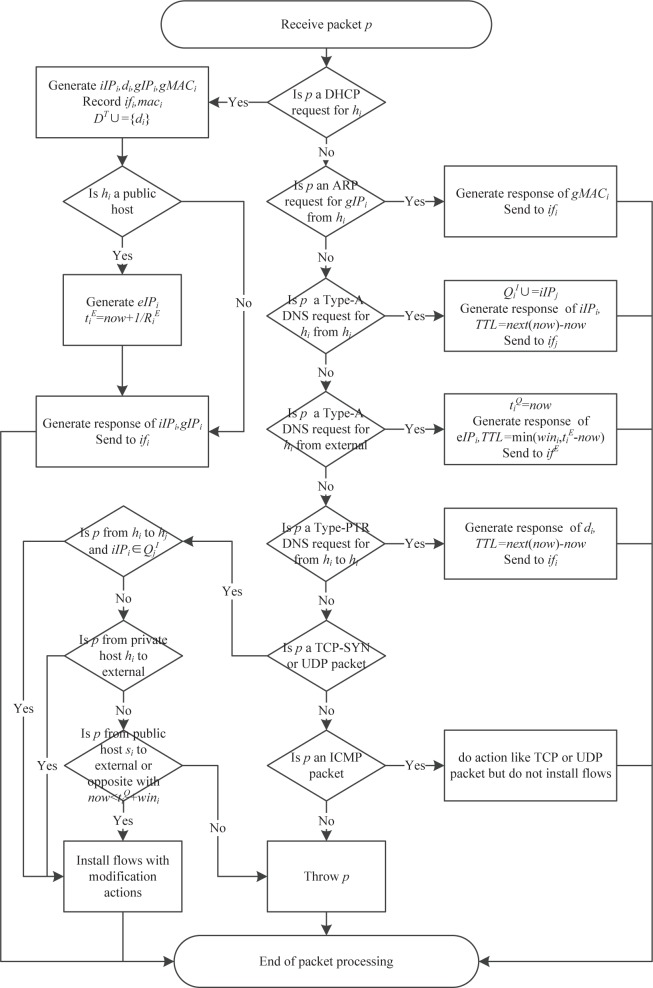
Flow chart of Algorithm 1.

The general packet processing algorithm is presented in Algorithm 1. The flows in the algorithm are the combined description of all software-defined switches in the path, in fact the action of the flows in the source and destination switches should only contain part of the modification action, and the intermediate switches should only contain the forward action. The flow chart of Algorithm 1 is presented in Fig 2.

**Algorithm 1** Packet Processing Algorithm

**for all** packets p **do**

    **if** p is a DHCP request for *h*_*i*_ **then**

        generate *iIP*_*i*_, *d*_*i*_, *gIP*_*i*_, *gMAC*_*i*_, record *if*_*i*_, *mac*_*i*_, *D*^*x*^∪ = {*d*_*i*_}

        **if** *h*_*i*_ is a public host **then**

            generate *eIP*_*i*_, $t_{i}^{E}=now+1/R_{i}^{E}$

        **end if**

            generate response of *iIP*_*i*_, *gIP*_*i*_ and send to *if*_*i*_

        **else if** p is an ARP request for *gIP*_*i*_ from *h*_*i*_ **then**

            generate response of *gMAC*_*i*_ and send to *if*_*i*_

        **else if** p is a Type-A DNS request for *h*_*i*_ from *h*_*j*_ **then**

            $Q_{i}^{I}=Q_{i}^{I}\cup \left\{iIP_{j}\right\}$

            generate response of *iIP*_*i*_,*TTL* = *next*(*now*)−*now* and send to *if*_*j*_

        **else if** p is a Type-A DNS request for *h*_*i*_ from external **then**

            $t_{i}^{Q}=now$, generate response of $eIP_{i},TTL=\min\left(win_{i},t_{i}^{E}-now\right)$ and send to *if*^*E*^

        **else if** p is a Type-PTR DNS request for *h*_*i*_ from *h*_*i*_ **then**

            generate response of *d*_*i*_ and send to *if*_*i*_

        **else if** p is a TCP-SYN or UDP **then**

            **if** p is from *h*_*i*_ to *h*_*j*_ and $iIP_{i}\in Q_{j}^{I}$ then

                install forward direction flows in switches with action

                    *p*.*srcMAC* ≔ *gMAC*_*j*_,*p*.*dstMAC* ≔ *mac*_*j*_,*Forward* → *if*_*j*_

                install backward direction flows in switches with action

                    *p*.*srcMAC* ≔ *gMAC*_*i*_,*p*.*dstMAC* ≔ *mac*_*i*_,*Forward* → *if*_*i*_

            **else if** p is from private host *h*_*i*_ to external **then**

                install forward direction flows in switches with action

                    *p*.*srcMAC* ≔ *gMAC*_*i*_,*p*.*dstMAC* ≔ GMAC,*Forward* → *if*^*E*^

                install backward direction flows in switches with action

                    *p*.*srcMAC* ≔ *gMAC*_*i*_,*p*.*dstMAC* ≔ *mac*_*i*_,*Forward* → *if*_*i*_

            **else if** p is from public host *s*_*i*_ to external or opposite with $now<t_{i}^{Q}+win_{i}$ **then**

                install outside direction flows in switches with action

                    *p*.*srcIP* ≔ *eIP*_*i*_,*p*.*srcMAC* ≔ *gMAC*_*i*_,*p*.*dstMAC* ≔ *GMAC*,*Forward* → *if*^*E*^

                install inside direction flows in switches with action

                    *p*.*dstIP* ≔ *iIP*_*i*_,*p*.*srcMAC* ≔ *gMAC*_*i*_,*p*.*dstMAC* ≔ *mac*_*i*_,*Forward* → *if*_*i*_

            **else**

                throw p

            **end if**

        **else if** p is a ICMP packet **then**

            **do** action like TCP or UDP packet but do not install flows

        **else**

            throw p

        **end if**

    **end** **for**

All throw packets, including error packets and connection failure packets, are recorded for subsequent dynamic mutation and attack behavior analysis. Error packets include: 1) packets with incorrect mapping of interfaces, MAC addresses, and IP addresses; and 2) TCP-SYN, UDP, and ICMP packets from an internal host to an external address. Connection failure packets include: 1)DNS packets querying currently unused internal domain names; 2) TCP-SYN, UDP, and ICMP packets connecting to currently unused internal or external addresses; 3) TCP-SYN, UDP, and ICMP packets from a source address that is an internal address and is not in the DNS query list of the destination host; and 4) TCP-SYN, UDP, and ICMP packets that the destination host is a public host, the source address is an external address, and the time is not in the time window of the destination host.

#### 4.2.2 Domain name and external address mutation algorithm

The domain names of all hosts are periodically changed at fixed times, and no assigned domain name will be repeated in *x* cycles; therefore, the mutation space is *S*^*D*^ − *D*^*x*^. The external addresses of all public hosts are changed randomly according to their mutation rate, and no external address that is assigned to mutated hosts can be the same as the external addresses of all non-mutated hosts; therefore, the mutation space is $S^{E}-\left\{eIP_{i}|s_{i}\in Servers\wedge t_{i}^{E}>now\right\}$. The general domain name and external address mutation algorithm is presented in Algorithm 2.

**Algorithm** 2 Mutation Algorithm

**for each** time of performing domain name mutation **do**

    **for each** *h*_*i*_ **do**

        random select *d*_*i*_ from *S*^*D*^−*D*^*x*^

        *D*^*x*^∪ = {*d*_*i*_}

    **end** **for**

    remove expired domain name from *D*^*x*^

**end** **for**

**for each** time of performing external address mutation **do**

    $A=\left\{eIP_{i}|s_{i}\in Servers\wedge t_{i}^{E}>now\right\}$

    **for each** *s*_*i*_ that $t_{i}^{E}\leq now$ **do**

        random select *eIP*_*i*_ from *S*^*E*^−*A*

        *A* ≔ *A*∪*eIP*_*i*_, $t_{i}^{E}=now+1/R_{i}^{E}$

    **end** **for**

**end** **for**

In the random selection of the domain name and external address in the above algorithm, we consider the following two methods: 1) uniform mutation, that is, the domain names and the external addresses are uniformly selected in the mutation space; and 2) failure-prior mutation, that is, the destination domain names and the addresses of the connection failure packets are preferentially selected.

### 4.3 Communication protocol

In RDAM, the client host must use the dynamic domain name of the server host to access the services it provides, and the client host can obtain the dynamic domain name of the server host in two ways: 1) for temporary services, the server host obtains its domain name through rDNS (nslookup) and informs the client host; or 2) for long-term services, the client host accesses the security authentication server to obtain the dynamic domain name list according to its authority, and finds the dynamic domain name of the server host in the list.

**Fig 3:**
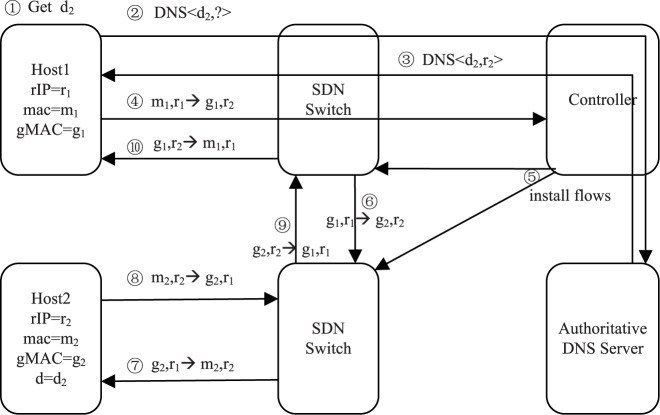
Communication between internal hosts.

Fig 3 shows the communication process between internal hosts. After obtaining the domain name of the server host, the client host sends a DNS query with the domain name, the controller and the DNS server response the server host’s internal address; the TTL value in the DNS response is the valid time of the current domain name; and the controller records the client host in the internal DNS query list (steps 1–3). The client host can then initiate the connection using the address of the server host, and the software-defined switch forwards the initial packet to the controller (step 4). The controller installs relevant flows in the software-defined switches in the path according to Algorithm 1 (step 5). Future packets of this connection will be modified and forwarded in software-defined switches according to the above installed flows in the flow table (steps 6–10).

**Fig 4:**
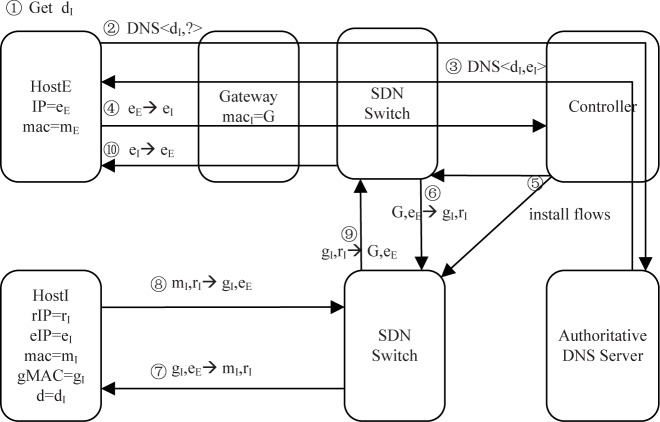
Communication of an external host requesting a public host’s service.

Fig 4 shows the communication process of an external host requesting a public host’s service. In this case, the client host obtains the external address of the server host through a DNS request; the TTL value in the DNS response is the minimum value of the valid time of the current external address and the time window; and the controller records the external DNS query time (steps 1–3). Similar to the above scenario, the initial packet of the client host is forwarded to the controller by a software-defined switch (step 4). According to Algorithm 1, after confirming the time window, the controller installs relevant flows in the software-defined switches in the path (step 5). The rest of the packets will be modified and forwarded in software-defined switches according to these flows (steps 6–10).

### 4.4 Additional techniques for robustness

In order to enhance the availability and security of the network, RDAM also used several other techniques to improve the network functionality and increase defense against reconnaissance attacks.

#### 4.4.1 Application level gateway service (ALG)

When an external host accesses a public host, the destination IP address is different from the actual IP address of the server host. However, in some special application protocols, such as file transfer protocol (FTP), generic routing encapsulation (GRE), etc., the payload contains the server host's IP address to establish connections. Therefore, IP address mutation may disrupt the normal communication of these protocols. RDAM implements application level gateway service (ALG) in the switch connected to the gateway to perform the detection and transformation of the payload when the internal address and the external address of the public host are interchanged, ensuring normal communication through the protocol without requiring the user to implement any special configuration.

#### 4.4.2 Fingerprint mutation

The attacker can use fingerprinting tools such as Nmap to identify the target host's fingerprint information, such as operating system, set of services, etc., for the purpose of finding a target host’s vulnerabilities. RDAM uses the recognition and replacement method to realize the confusion of some fingerprint information [33], including the version of the server software in FTP and the hypertext transfer protocol (HTTP), and the operating system version.

## Implementation and evaluation

In order to investigate and validate the feasibility of RDAM, we implemented a RDAM prototype and deployed it in our laboratory network according to Fig 1, in which all the software-defined switches and the controller were implemented in the X86 platform using Intel's DPDK (Data Plane Development Kit) [34]. The network's external address space was a Class C subnet, and there were 36 hosts in the network distributed within three physical subnets. One subnet included a web server and a FTP server, which were both set as public hosts, while in the other subnets, four internal hosts enabled the remote desktop service, of which two hosts were set as public hosts.

We ran several network activities during mutation, including: 1) web browsing, FTP file download, and remote desktop connections of internal hosts and external hosts to public hosts; 2) remote desktop connections of internal hosts to private hosts; and 3) connection of internal hosts to the Internet. All network activities behaved normally, and long-lived connections were maintained during mutations. The network quickly completed the DNS responses and the establishment of connections, while the established data flow packet transmission delay was negligible. Our implementation proved that RDAM is feasible in real networks.

In the following, we will present the defense performance of RDAM against scanning attacks and worm propagation, as well as its cost, through theoretical analysis and simulation experiments.

### 5.1 Scanning attack

Scanning is the primary step in most network attacks. The attacker uses scanning tools, such as Nmap, to discover the active hosts in the target network, and then carries out further attacks. In this paper, we consider the following two typical scanning strategies: 1) non-repeat scans, which never scan the same address twice, usually employed in scanning tools; and 2) repeatable scans, in which the addresses scanned are generated randomly so they may be repeated, usually employed in worms and other malwares. In the following, we will analyze the defense performance of RDAM when using uniform mutation and failure-prior mutation by calculating the expected ratio of hosts missed by the scanner (to total hosts).

#### 5.1.1 Repeatable scans

In repeatable scans, the address or domain name scanned is randomly generated. Therefore, regardless of which mutation method the defender uses, the probability of the scanner hitting a designated host is the same as the probability in the static address environment.

Here, we analyze repeatable scanning of domain names and IP addresses from internal and external scanners in RDAM.

(1) Domain name scans: First, consider that the internal/external scanner scans domain names. Because the size of the domain name space is *m*^*D*^, the probability of the scanner hitting each host in each scan is 1/*m*^*D*^. Then, in *k* scans the scanner misses each host with probability (1−1/*m*^*D*^)^*k*^. Because the probability of each host being hit is independent of each other host, the probability of the scanner missing each host is also the expected ratio of missed hosts. Therefore, the expected ratio of missed hosts in *k* scans is

$$P_{miss}\left(k\right)={\left(1-1/m^{D}\right)}^{k}.$$

Thus, when the number of scans is the same as the external address space size *m*^*E*^, RDAM allows ${\left(1-1/m^{D}\right)}^{m^{E}}\approx e^{-m^{E}/m^{D}}$ of the hosts to be missed by the scanner.

(2) IP address scans by an external scanner: In RDAM, the probability that each public host can be accessed through its external address is *p*, so the probability of the scanner hitting each host in each scan is *p*/*m*^*E*^. Therefore, the expected ratio of missed hosts in *k* scans is

$$P_{miss}\left(k\right)={\left(1-p/m^{E}\right)}^{k}$$

Thus, when the number of scans is *m*^*E*^, RDAM allows ${\left(1-p/m^{E}\right)}^{m^{E}}\approx e^{-p}$ of the hosts to be missed by the scanner.

(3) IP address scans by an internal scanner: In RDAM, internal hosts can access another host only when they are in the internal DNS query list; therefore, the internal scanner cannot hit any host by scanning IP addresses, and the expected ratio of missed hosts in *k* scans is

$$P_{miss}\left(k\right)=1.$$

**Fig 5:**
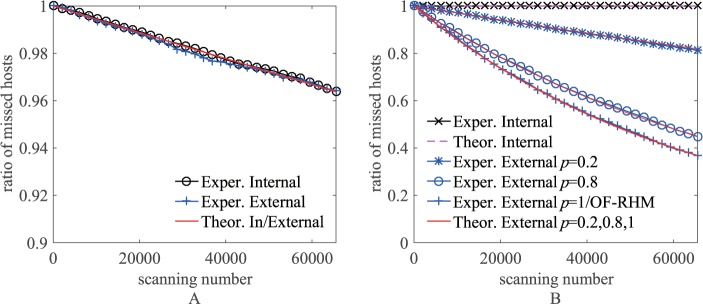
The ratio of hosts missed by repeatable scanners. (A) Scan domain names. (B) Scan IP addresses.

To perform simulation experiments, we created a network of SDN switches using Mininet [35], and developed the RDAM controller using POX [36] to manage the SDN network. The network included *n*^*I*^ = 12000 internal hosts, of which *n*^*E*^ = 6000 hosts were public hosts; the external address space was a Class B subnet (*m*^*E*^ = 65536); and the length of the 3LD was 4 (*l* = 4). Fig 5 shows the theoretical and experimental results of the ratio of missed hosts by the internal and external repeatable scanners scanning domain names and IP addresses as the scanning number increases. In the simulation, every data point was taken as the average of 10 runs, and the scanner executed a total of *m*^*E*^ scans. In existing network address shuffling techniques, such as NASR [10], OF-RHM [11], etc., internal and external scanners can directly access an internal host once they hit the host’s IP address, because of the lack of the DNS query list and the time window methods. Therefore, the result of the internal/external scanner scanning IP addresses in OF-RHM is equivalent to the result of the external scanner scanning IP addresses in RDAM when *p* = 1 in Fig 5(B).

From the figure, we can see that the experimental results are basically the same as those predicted by the theoretical analysis. RDAM allows 96.2% of hosts to be missed by internal and external scanners scanning domain names; an internal scanner cannot hit any host by scanning IP addresses; for external scanners scanning IP addresses, when *p* = 1, that is, no time window, RDAM allows only 37% of hosts to be missed, while when *p* = 0.2, RDAM allows 81% of hosts to be missed by adjusting the time window setting.

#### 5.1.2 Non-repeat scans in uniform mutation

Assume that *r* denotes the ratio of the attacker scanning rate to the defender mutation rate; that the external address mutation rate of all public hosts is the same; and that they mutate at the same time. When *r* ≤ 1, the domain name (IP address) will be changed between any two scans; therefore, the scanning process is equivalent to a repeatable scan, and the scan results are consistent with those presented in the previous section.

Next, we analyze the case when *r* ≥ 1, assuming *r* is an integer.

(1) Domain name scans: First, consider that the internal/external scanners scan domain names. Because the hosts’ domain names are uniformly distributed in the domain name space after each mutation, between two mutations, the probability of the scanner hitting each host in *k*′ scan is *k*′/*m*^*D*^. *k* ≤ *m*^*D*^ scans can be divided into ⌊*k*/*r*⌋+1 mutations; therefore, the expected ratio of missed hosts in *k* scans, which is also the probability of the scanner missing each host, is

$$P_{miss}\left(r,k\right)=\left(1-\left(k-\left\lfloor k/r\right\rfloor r\right)/m^{D}\right){\left(1-r/m^{D}\right)}^{\left\lfloor k/r\right\rfloor }.$$

Thus, we can see that *P*_*miss*_(*r*,*k*) decreases as *r* increases, and

1) when *r* ≪ *k*, $P_{miss}\left(r,k\right)\approx {\left(1-r/m^{D}\right)}^{\left\lfloor k/r\right\rfloor }\approx {\left(1-r/m^{D}\right)}^{k/r}\approx e^{-k/m^{D}}$, *P*_*miss*_(*r*,*k*) reaches its maximum, and RDAM achieves the best defense performance;

2) when *r* < *k* ∧ *r* → *k*, *P*_*miss*_(*k*) ≈ 1−*r*/*m*^*D*^; and

3) when *r* ≥ *k*, *P*_*miss*_(*r*,*k*) ≈ 1−*r*/*m*^*D*^, the domain name mutation offers no additional defense performance compared to the static environment.

From the above analysis we can obtain $1-m^{E}/m^{D}\leq P_{miss}\left(r,m^{E}\right)\leq e^{-m^{E}/m^{D}}$. Because *m*^*E*^ ≪ *m*^*D*^, so $e^{-m^{E}/m^{D}}\approx 1-m^{E}/m^{D}$. Therefore, when the number of scans is *m*^*E*^, RDAM allows 1−*m*^*E*^/*m*^*D*^ of the hosts to be missed by the scanner.

(2) IP address scans by an external scanner: In RDAM, the probability that each public host can be accessed through its external address is *p*. Therefore, the expected ratio of missed hosts in *k* scans is

$$P_{miss}\left(r,k\right)=\left(1-p\left(k-\left\lfloor k/r\right\rfloor r\right)/m^{E}\right){\left(1-pr/m^{E}\right)}^{\left\lfloor k/r\right\rfloor }.$$

Thus, we can obtain 1−*p*≤*P*_*miss*_(*r*,*m*^*E*^) ≤ *e*^−*p*^. Therefore, when the number of scans is *m*^*E*^, RDAM allows at most *e*^−*p*^ and at least 1−*p* of the hosts to be missed by the scanner.

(3) IP address scans by an internal scanner: In RDAM, the internal scanner cannot hit any host by scanning IP addresses. Therefore, the expected ratio of missed hosts in *k* scans is

$$P_{miss}\left(r,k\right)=1$$

**Fig 6:**
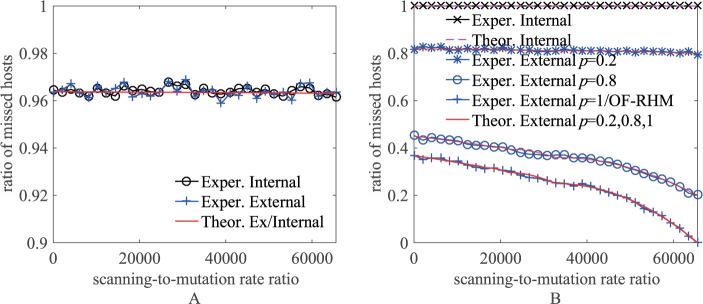
The ratio of hosts missed by non-repeat scanners in uniform mutation. (A) Scan domain names. (B) Scan IP addresses.

Fig 6 shows the theoretical and experimental results of the ratio of hosts missed by the internal and external non-repeat scanners scanning domain names and IP addresses when the defender adopts uniform mutation as *r* increases. The simulation environment is the same as that described in Section 5.1.1. In the simulation, every data point was taken as the average of 10 runs, and the scanner executed a total of *m*^*E*^ scans. In particular, the result of the internal/external scanner scanning IP addresses in OF-RHM when the defender adopts uniform mutation is equivalent to the result of the external scanner scanning IP addresses in RDAM when *p* = 1 in Fig 6(B).

From the figure, we can see that the experimental results are basically the same as those predicted by the theoretical analysis. RDAM allows 96.2% of hosts to be missed by internal and external scanners scanning domain names; internal scanners cannot hit any host by scanning IP addresses; for external scanners scanning IP addresses, when *p* = 1, that is, no time window, RDAM allows at most 37% of hosts to be missed, and the ratio decreases as *r* increases, while when *p* = 0.2, RDAM allows 80% of hosts to be missed by adjusting the time window setting.

#### 5.1.3 Non-repeat scans in failure-prior mutation

Assume that *r* denotes the ratio of the attacker scanning rate to the defender mutation rate; that the external address mutation rate of all public hosts is the same; and that they mutate at the same time. Similarly, when *r* ≤ 1, the scan results are consistent with those presented in Section 5.1.1.

Next, we also analyze the case when *r* ≥ 1, assuming *r* is an integer.

(1) Domain name scans: First, consider that the internal/external scanner scans domain names, and denote the number of hosts as *n* (*n* = *n*^*I*^ for the internal scanner and *n* = *n*^*E*^ for the external scanner). In failure-prior mutation, the domain names missed by the scanner are preferentially selected as hosts’ domain names, and the scanner will never scan those domain names in non-repeat scans, so some hosts will not be hit by the scanner. Denote *X*_*i*_ as the number of inaccessible hosts in the *i*^th^ mutation interval; then *X*_1_ = 0.

In the *i*^*th*^ mutation interval, the domain names of the remaining *n*−*X*_*i*_ hosts are uniformly distributed in the domain name space, the size of which is *m*^*D*^−*X*_*i*_. In this interval, the probability of the scanner hitting each host in *k*′ scans is $\frac{n-X_{i}}{n}\frac{k^{\prime }}{m^{D}-X_{i}}$, and the scanning number of hitting a host follows an hypergeometric distribution; thus, the expected hitting number is *k*′(*n*−*X*_*i*_)/(*m*^*D*^−*X*_*i*_). The scanner can execute *r* scans in this interval, and therefore the number of missed domain names is *r*−*r*(*n*−*X*_*i*_)/(*m*^*D*^−*X*_*i*_), which is the number of inaccessible hosts in the *i+1*^*th*^ mutation interval. Considering that the number of hosts is *n*, we can obtain *X*_*i*+1_ = min(*n*,*r*−*r*(*n*−*X*_*i*_)/(*m*^*D*^−*X*_*i*_)).

*k* ≤ *m*^*D*^ scans could be divided into ⌊*k*/*r*⌋ + 1 mutations; therefore, the expected ratio of missed hosts in *k* scans, which is also the probability of the scanner missing each host, is

$$P_{miss}\left(r,k\right)=\left(1-\left(k-\left\lfloor k/r\right\rfloor r\right)\frac{1-X_{\left\lfloor k/r\right\rfloor +1}/n}{m^{D}-X_{\left\lfloor k/r\right\rfloor +1}}\right)\prod_{1\leq i\leq \left\lfloor k/r\right\rfloor }\left(1-r\frac{1-X_{i}/n}{m^{D}-X_{i}}\right)$$

where *X*_1_ = 0, *X*_*i*+1_ = min(*n*,*r*−*r*(*n*−*X*_*i*_)/(*m*^*D*^−*X*_*i*_)).

Thus we can obtain that

1) when *r* ≪ *k* ∧ *r* ≪ *n*, *X*_*i*_ is negligible, so $P_{miss}\left(r,k\right)\approx e^{-k/m^{D}}$;

2) when *r* < *k* ∧ *X*_*i*_ < *n*, the larger *r* is, the larger *X*_*i*_ is, and the better the defense performance is, so *P*_*miss*_(*r*,*k*) increases as *r* increases;

3) when *r* < *k* ∧ *X*_2_ = *n*, namely, *n*/(1−*n*/*m*^*D*^) ≤ *r* < *k*, *X*_*i*_ = *n*,*i* > 1, we can obtain *P*_*miss*_(*r*,*k*) ≈ 1−*r*/*m*^*D*^, so *P*_*miss*_(*r*,*k*) decreases as *r* increases; and

4) when *r* ≥ *k*, *P*_*miss*_(*r*,*k*) ≈ 1−*k*/*m*^*D*^.

From the above analysis we find that when *r* = *n*/(1−*n*/*m*^*D*^), *P*_*miss*_(*r*,*k*) reaches its maximum; when *r* ≥ *k*, *P*_*miss*_(*r*,*k*) reaches its minimum, so $1-k/m^{D}\leq P_{miss}\left(r,k\right)\leq 1-\frac{n/\left(1-n/m^{D}\right)}{m^{D}}=\frac{m^{D}-2n}{m^{D}-n}$. Therefore, when the number of scans is *m*^*E*^, RDAM allows at most $\frac{m^{D}-2n}{m^{D}-n}$ and at least 1−*m*^*E*^/*m*^*D*^ of the hosts to be missed by the scanner.

(2) IP address scans by an external scanner: In RDAM, the probability that each public host can be accessed through its external address is *p*. Therefore, the expected ratio of missed hosts in *k* scans is

$$P_{miss}\left(r,k\right)=\left(1-p\left(k-\left\lfloor k/r\right\rfloor r\right)\frac{1-X_{\left\lfloor k/r\right\rfloor +1}/n^{E}}{m^{E}-X_{\left\lfloor k/r\right\rfloor +1}}\right)\prod_{1\leq i\leq \left\lfloor k/r\right\rfloor }\left(1-pr\frac{1-X_{i}/n^{E}}{m^{E}-X_{i}}\right)$$

where *X*_1_ = 0, *X*_*i*+1_ = min(*n*^*E*^,*r* − *pr*(*n*^*E*^ − *X*_*i*_)/(*m*^*E*^ − *X*_*i*_)).

Similarly, we find that when *r* < *k* ∧ *X*_2_ = *n*, namely *n*/(1−*pn*^*E*^/*m*^*E*^) ≤ *r* < *k*, *P*_*miss*_(*r*,*k*) ≈ 1−*pr*/*m*^*E*^ can reach its maximum; when *r* ≥ *k*, *P*_*miss*_(*r*,*k*) ≈ 1−*pk*/*m*^*D*^ reaches its minimum, so $1-p\leq P_{miss}\left(r,m^{E}\right)\leq \frac{m^{E}-2pn^{E}}{m^{E}-pn^{E}}$. Therefore, when the number of scans is *m*^*E*^, RDAM allows at most $\frac{m^{E}-2pn^{E}}{m^{E}-pn^{E}}$ and at least 1−*p* of the hosts missed to be by the scanner.

(3) IP address scans by an internal scanner: In RDAM, the internal scanner cannot hit any host by scanning IP addresses. Therefore, the expected ratio of missed hosts in *k* scans is

$$P_{miss}\left(r,k\right)=1$$

**Fig 7:**
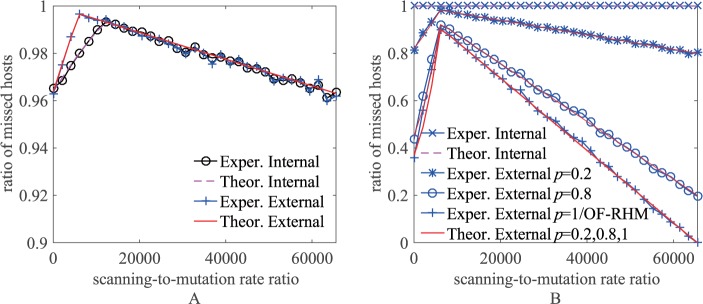
The ratio of hosts missed by non-repeat scanners in failure-prior mutation. (A) Scan domain names. (B) Scan IP addresses.

Fig 7 shows the theoretical and experimental results of the ratio of hosts missed by the internal and external non-repeat scanners scanning domain names and IP addresses when the defender adopts failure-prior mutation as *r* increases. The simulation environment is the same as that presented in Section 5.1.1. In the simulation, every data point was taken as the average of 10 runs, and the scanner executed a total of *m*^*E*^ scans. In particular, the result of the internal/external scanner scanning IP addresses in OF-RHM when the defender adopts failure-prior mutation is equivalent to the result of the external scanner scanning IP addresses in RDAM when *p* = 1 in Fig 7(B).

From the figure, we can see that the experimental results are basically the same as those predicted by the theoretical analysis. For the internal scanner (external scanner) scanning domain names, the ratio of missed hosts first increases then decreases as *r* increases, and RDAM allows at most 99.7% (99.3%) and at least 96.2% of hosts to be missed. The internal scanner cannot hit any host by scanning IP addresses. For the external scanner scanning IP addresses, the ratio of missed hosts also first increases then decreases as *r* increases; when *p* = 1, that is, no time window, RDAM allows at most 89.9% of hosts to be missed, while when *p* = 0.2, RDAM allows at most 98.1% and at least 80% of hosts to be missed by adjusting the time window setting.

Through the analysis of the above three cases, we can see that RDAM increases the scanning space of the scanner through the dynamic domain name method, and reduces the probability that the scanner can access hosts by scanning the IP address through the DNS query list and time window. RDAM offers better defense performance than the general network address shuffling method, whether it is for the repeatable scanning attack or the non-repeat scanning attack.

### 5.2 Worm propagation

Computer worms are programs that self-propagate across a network, exploiting security or policy vulnerabilities in widely used services. “Scan-based worms” propagate by scanning random IP addresses and infecting active vulnerable targets, which need no human activation and thus can propagate much faster than “email worms” [37]. Many well-known worms, such as Code Red [38], Blaster [39], Witty worm [40], Conficker [41], Storm [42], etc., are all scan-based worms. In this paper, we consider worms based on IP address and domain name scanning, even though there are no known worms based on domain name scanning currently [43].

**Fig 8:**
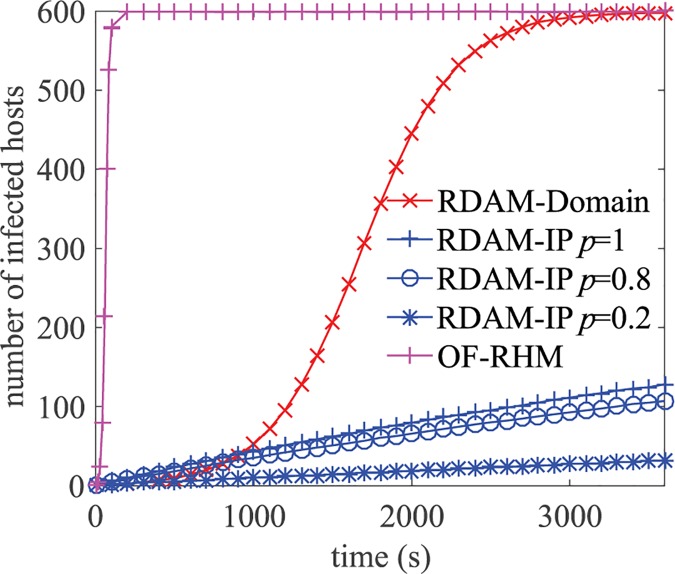
Propagation of worms based on IP address and domain name scanning.

In this paper, we conducted experiments on worm propagation in the same simulation environment as in Section 5.1.1. Worm propagation behavior was experimented using a worm daemon [44], which consists of a client module and a server module. The client module is used to send “infection” datagrams to scanned targets in the target space on a specific UDP port; the server module is used to listen on the UDP port, and start the client module after receiving an “infection” datagram. The target space of the worm based on IP address scanning is the external address space, and the target space of the worm based on domain name scanning is the domain name space. In RDAM, the domain names and external addresses of all hosts are uniformly distributed, and therefore the attacker can obtain no information about a non-uniform vulnerable-host distribution; the best advantage the attacker can gain from a uniform distribution is to use uniform scanning, that is, randomly selecting targets in the target space [45]. Therefore, in the experiment, the worms use uniform scanning strategy. Fig 8 shows the propagation of worms based on IP address and domain name scanning in RDAM, and the propagation of the worm based on IP address scanning when all internal hosts dynamically mutate in the external address space using OF-RHM. The results in the figure were all taken from the averages of five experimental results. In each experiment, we ran server modules in 5% of the internal hosts and initially started two client modules in a public host and an external host, respectively; the worm scanning speed was 10 times/s, and each experiment lasted 1 hour.

From the experiments and above analyses, we can see that worms based on IP address scanning have the following characteristics in RDAM: 1) worms cannot propagate within internal hosts because internal hosts cannot directly access each other by scanning IP addresses; and 2) worms cannot infect private hosts because external infected hosts cannot access the private host and internal hosts cannot infect each other. Therefore, worms based on IP address scanning in RDAM are equivalent to external repeatable scanning attacks scanning external address spaces. Following the analysis in Section 5.1.1, RDAM can effectively reduce the ratio of infected public hosts through adjustments to the time window, and from Fig 8, we can see that the propagation results of worms based on IP address scanning in RDAM are consistent with the results shown in Fig 5. In summary, RDAM can effectively defend against worms based on IP address scanning.

With regard to worms based on domain name scanning, an attacker who already knows the domain name space of RDAM can spread a worm based on domain name scanning by setting the domain name scanning space in the worm. However, the space can be easily changed by re-configuration. In addition, according to [46], the propagation time of a uniform scan worm is proportional to the size of the scanning space. Therefore, RDAM can greatly increase the propagation time of worms based on domain name scanning, and from Fig 8, we can see that the propagation of the worm based on domain name scanning is significantly slower than the propagation of the worm based on IP address scanning in OFRHM. In summary, RDAM can effectively defend against worms based on domain name scanning.

### 5.3 Overhead

The space overhead of RDAM mainly includes the size of the flow table. The time overhead of RDAM mainly includes the delay from the DNS request to establish a connection and the delay resulting from packet modification and forwarding in software-defined switches.

#### 5.3.1 Flow table size

**Fig 9:**
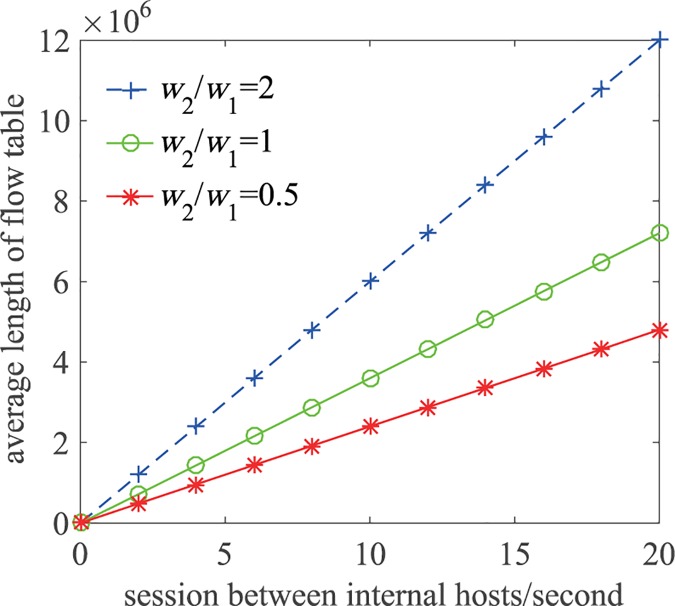
Flow table lengths for different session establishment rates.

In RDAM, each TCP/UDP session needs to establish two flow table entries in the software-defined switches in the path. Assume in RDAM, each internal host establishes on average *w*_1_ sessions with other internal hosts per second, *w*_2_ sessions with external hosts per second, and each session on average takes *θ* seconds to terminate. Then according to Little’s law in queuing theory, the mean length of the flow table is (*w*_1_ + 2*w*_2_)*n*^*I*^*θ*. Fig 9 shows the flow table lengths for various session establishment rates (*w*_1_ and *w*_2_/*w*_1_) when *n*^*I*^ = 12000, *θ* = 10*s*.

#### 5.3.2 Operational delay

We tested the operational delay caused by RDAM in our laboratory prototype. The software-defined switches and the controller were implemented in the X86 platform operating at 4 GHz using Intel's DPDK; the controller had two cores and 4 GB of memory; the switches had six cores and 8 GB of memory. All internal hosts, the DNS server, the DHCP server, and the security authentication server had two cores operating at 3.7 GHz and 4 GB of memory; the internal hosts ran the Win7 operating system, and the servers ran the Windows Server 2003 operating system. External hosts were generated through a server running Hyper-V with 32 cores operating at 3.9 GHz and 64 GB of memory; each external host had one core and 512 MB of memory running the Windows XP operating system. We tested 3 scenarios: scenarios with no flow table entry and 1 million flow table entries in the switches, and a scenario without RDAM, whereby hosts access each other using IP addresses through common switches. In each scenario, we measured the delay results of connections between internal hosts and connections of external hosts accessing public hosts, ran 100 trials respectively by using 25 internal hosts/external hosts visiting 4 public hosts. Table 1 shows the experimental results of the connection delay (the delay from the DNS request to establish a connection) and the transmission delay (the delay resulting from packet processing in software-defined switches after establishing the connection). From the results, we can see that the connection delay is unlikely to be noticed by users, and the transmission delay is negligible compared with the whole packet transmission process.

**Table 1:** Operation delay with and without RDAM.

	Connections between internal hosts		Connections of external hosts accessing public hosts	
	Connection delay	Transmission delay	Connection delay	Transmission delay
Without RDAM	0	<1ms^+^	0	<1ms
RDAM with 0 flow table entry	24.57ms(1.52ms)*	<1ms	26.34ms(1.73ms)*	<1ms
RDAM with 1 million flow table entries	25.42ms(1.62ms)*	<1ms	26.72ms(1.96ms)*	<1ms

^+^The unit “ms” is the abbreviation for “millisecond”.

*average delay (standard deviation)

### 5.4 Discussion

RDAM is a network-based defense method, so it can be used simultaneously with host-based defense methods, such as firewall, to strengthen the network security. Also, RDAM does not affect the normal use of VPN (Virtual Private Network), because 1) when a VPN connection is established, the corresponding flow table entries are installed in the SDN switches, so VPN communications will not be interrupted due to domain name and network address mutation, and 2) as we described in Section 4.4.1, RDAM implements ALG in the switch connected to the gateway to perform the detection and address information replacement of the TCP/UDP payloads including those of VPN protocols.

The topology of RDAM is that a number of hosts and physical subnets are connected to the SDN network composed of SDN switches and the controller. However, RDAM does not support the topology that hosts and physical subnets are connected to the SDN network behind routers, because communications within routers will not be forwarded to the controller (if they can, the routers are actually SDN switches), and thus will not be protected by RDAM.

In addition, because internal hosts cannot access each other by scanning IP addresses, so some LAN software based on scanning IP addresses cannot work normally in RDAM. In further work, we will consider establishing a virtual network in RDAM, mapping the IP addresses of the hosts using LAN software to the network to allow these hosts to communicate through a specific port and protocol without leaking the IP addresses.

## Conclusion

In this paper, we presented a defense technique based on assigning random domain names and network addresses, called RDAM, which defends against adversarial reconnaissance and scanning by dynamically mutating domain names and IP addresses. In RDAM, both the domain name and network address are changed dynamically, and an internal host can access other internal hosts only when it is in the DNS query list, and an external host can access a public host only when it is in the public host's time window. In addition, all internal hosts occupy different subnets and communicate through their virtual gateways.

We have presented the basic architecture and communication protocols in this paper, and evaluated the defense performance against scanning attacks and worm propagation, as well as the overhead, through theoretical analysis and experiment. Our evaluation and experimentation show that RDAM can effectively defend against scanning attacks and worm propagation by increasing the scanning space of the scanner through the dynamic domain name method, and reducing the probability that the scanner can access hosts by scanning the IP address through the DNS query list and time window, while satisfying practical operation overhead demand.
